# Characterizing the postnatal hypothalamic–pituitary–adrenal axis response of in utero heat stressed pigs at 10 and 15 weeks of age

**DOI:** 10.1038/s41598-021-01889-w

**Published:** 2021-11-18

**Authors:** Jacob M. Maskal, Luiz F. Brito, Alan W. Duttlinger, Kouassi R. Kpodo, Betty R. McConn, Christopher J. Byrd, Brian T. Richert, Jeremy N. Marchant, Donald C. Lay, Shelbi D. Perry, Matthew C. Lucy, Tim J. Safranski, Jay S. Johnson

**Affiliations:** 1 Department of Animal Sciences, Purdue University, West Lafayette, IN 47907 USA; 2 Oak Ridge Institute for Science and Education, Oak Ridge, TN 37830 USA; 3 Livestock Behavior Research Unit, USDA-ARS, West Lafayette, IN 47907 USA; 4 Division of Animal Sciences, University of Missouri, Columbia, MO 65221 USA

**Keywords:** Reprogramming, Neurophysiology, Agricultural genetics

## Abstract

In utero heat stress alters postnatal physiological and behavioral stress responses in pigs. However, the mechanisms underlying these alterations have not been determined. The study objective was to characterize the postnatal hypothalamic–pituitary–adrenal axis response of in utero heat-stressed pigs. Pigs were subjected to a dexamethasone suppression test followed by a corticotrophin releasing hormone challenge at 10 and 15 weeks of age. Following the challenge, hypothalamic, pituitary, and adrenal tissues were collected from all pigs for mRNA abundance analyses. At 10 weeks of age, in utero heat-stressed pigs had a reduced (*P* < 0.05) cortisol response to the corticotrophin releasing hormone challenge versus controls. Additionally, the cortisol response tended to be greater overall (*P* < 0.10) in 15 versus 10-week-old pigs in response to the dexamethasone suppression test. The cortisol response tended to be reduced overall (*P* < 0.10) in 15 versus 10-week-old pigs in response to the corticotrophin releasing hormone challenge. Hypothalamic corticotropin releasing hormone mRNA abundance tended to be greater (*P* < 0.10) in in utero heat-stressed versus control pigs at 15-weeks of age. In summary, in utero heat stress altered some aspects of the hypothalamic–pituitary–adrenal axis related to corticotropin releasing hormone signaling, and age influenced this response.

## Introduction

In utero heat stress (IUHS) alters the postnatal physiological and behavioral stress response of pigs^1,2^. Specifically, during exposure to acute stressors (e.g., birth, social stress, handling stress, weaning), an increase in circulating and salivary cortisol^3–5^ and adrenocorticotropic hormone (ACTH)^6^ have been reported for IUHS pigs when compared to in utero thermoneutral (IUTN) pigs. Interestingly, the opposite is true following more prolonged stressors (e.g., transport), whereby circulating cortisol levels are reduced in IUHS compared to IUTN pigs^7,8^ . In addition to hormonal influences, IUHS increases the incidence of stress-associated behaviors when pigs are exposed to common postnatal production stressors (e.g., weaning and/or transport)^5,7^. However, although the aforementioned studies^3–8^ have been instrumental in identifying how IUHS can impact postnatal measures of swine stress and welfare, none to our knowledge have attempted to elucidate mechanisms that underlie these responses.

The hypothalamic–pituitary–adrenal (HPA) axis plays an integral role in regulating the physiological and behavioral stress response in animals^9^. Its activation in response to stressors causes the release of corticotrophin-releasing hormone (CRH) from the hypothalamus, which binds to corticotropin-releasing hormone receptors (CRHR) in the anterior pituitary^9^. This activates the production of proopiomelanocortin (POMC), which is then converted into ACTH and released into circulation^10^. Adrenocorticotropin hormone then binds to melanocortin 2 receptors (MC2R) in the adrenal glands and stimulates glucocorticoid (i.e., cortisol) secretion^9^. Following secretion, glucocorticoids are dispersed throughout the body and bind to ubiquitously expressed glucocorticoid receptors (GR) to stimulate multiple responses to stress^11^. Responses mediated by this mechanism include regulation of inflammation^12^, gluconeogenesis^13^, and aggression^14^, among a myriad of other responses^11^. Under normal conditions, glucocorticoids also stimulate negative feedback by binding to GR in the hypothalamus and anterior pituitary to downregulate production of both CRH and ACTH, respectively, which ultimately downregulates peripheral responses to stress^15^.

Previous research in pig prenatal stress models have observed altered postnatal HPA axis function in offspring, which has been linked to altered postnatal physiological and behavioral stress responses^16–18^. As previously mentioned, IUHS causes alterations in postnatal physiological and behavioral stress responses of pigs^3–8^, yet to our knowledge there has not been research conducted to describe HPA axis function in these models. Additionally, IUHS research that has observed altered postnatal physiological and behavioral stress responses has been conducted in relatively young pigs that have not been given time to mature^3,5–7^, possibly providing time for development of physiology capable of adequately dealing with stressors. Previous studies describing development of mature circadian rhythms have only observed a true rhythm at approximately 3 to 4 months of age^19^, of which previously described IUHS studies only observed effects up to approximately 2 months of age^3–8^. In this context, it is necessary to investigate whether or not the onset of matured mechanisms controlling cortisol release play a significant role in regulating stress responses in growing pigs. Our hypothesis is that IUHS-induced changes in postnatal physiological and behavioral stress responses are driven by alterations to the HPA axis functionality that are also influenced by advancing age. Therefore, the study objective was to characterize the postnatal HPA axis response of IUHS and IUTN pigs at two different ages (10 and 15 weeks of age).

## Materials and methods

### Gestational procedures

The University of Missouri Animal Care and Use Committee approved all procedures involving pregnant pigs and their offspring (protocol # 9340) and animal care and use standards were based upon the *Guide for the Care and Use of Agricultural Animals in Research and Teaching*^20^. Additionally, the Animal Research: Reporting of In Vivo Experiments (ARRIVE) guidelines were adhered to. A full description of gestational and pre-weaning procedures for pigs included in this study along with all gestational (e.g., thermoregulatory data, gestation length, etc.) and pre-weaning litter data (e.g., piglet birth weights, live born piglets, piglets weaned, etc.) have been previously presented^8^. Briefly, 24 unrelated pregnant female pigs that had not previously given birth were evenly assigned to either a thermoneutral [TN; *n* = 12 pregnant pigs; 17.5 ± 2.1 °C; 70.2 ± 8.8% relative humidity (RH)]) or heat stress (HS; *n* = 12 pregnant pigs) chamber from day 0 to 59 of gestation. As previously described^8^, pregnant female pigs in the HS group were exposed to 17.0 ± 0.1 °C and 79.2 ± 10.1% RH from days 0 to 5 of gestation. Cyclic HS temperatures of 26.3 ± 3.0 °C nighttime to 31.4 ± 2.9 °C daytime and 61.2 ± 21.2% RH were then applied from days 6 to 10 of gestation. Finally, pregnant female pigs were exposed to cyclic HS temperatures of 28.4 ± 0.2 °C nighttime to 35.8 ± 0.2 °C daytime and 80.9 ± 6.0% RH from days 11 to 59 of gestation. During the 59-day thermal treatment phase, rectal temperature, respiration rate, and shoulder and ear skin temperatures were measured daily on all pregnant pigs at 0800 and 1500 h as previously described^8^. As previously reported^8^, all thermal measures were increased overall (*P* < 0.01) in HS when compared to TN-exposed pregnant pigs and feed intake was similar (*P* > 0.05) because all pregnant pigs were limit-fed per normal commercial production practices^21^. Following the 59-day thermal treatment period, all pregnant pigs were exposed to the same TN conditions for the remainder of gestation as previously described^8^. All litters were housed in accordance with the recommendations of the *Guide for the Care and Use of Agricultural Animals in Research and Teaching* (26 to 32 °C with heat lamps) from birth to weaning, and the environmental conditions for litters were the same, regardless of gestational treatment^8,20^. All pigs were weaned at 16.2 ± 0.4 days of age and transported from the University of Missouri swine farm located in Columbia, MO, USA to the Purdue University swine farm located in West Lafayette, IN, USA^8^.

### Postnatal procedures

The Purdue University Animal Care and Use Committee approved all postnatal animal procedures (protocol #1806001756) and animal care and use standards were based upon the *Guide for the Care and Use of Agricultural Animals in Research and Teaching*^20^. Additionally, the Animal Research: Reporting of In Vivo Experiments (ARRIVE) guidelines were adhered to. From weaning until 35 days of age, pigs were maintained in group pens as previously described in Maskal et al.^8^. Following the nursery phase testing (up to 35 days of age)^8^, pigs were group housed at 4 pigs/pen (1.08 m^2^/pig) in TN conditions at the Purdue University swine farm and TN temperatures were based on pig age^20^. Feed and water were provided ad libitum and diets (primarily corn and soybean meal) were formulated to meet or exceed the nutrient requirements based on pig age^21^. At 69.7 ± 1.3 days of age (10 weeks) and 106.5 ± 0.9 days of age (15 weeks), 48 pigs (*n* = 24 pigs per age category), balanced by in utero treatment and sex, were selected for HPA axis testing in two repetitions. Litter of origin was considered during animal selection to distribute pigs of separate litters as evenly as possible (*n* = 9 IUTN and 9 IUHS litters). All nursery phase data (e.g., diet composition, growth performance, blood characteristics, behavioral data) are available and previously described in detail^8^.

### HPA axis testing

The HPA axis functionality was tested using a dexamethasone (DEX) suppression test followed by a corticotrophin-releasing-hormone (CRH) challenge over a 2-day period using procedures previously validated and described in pigs^22,23^. The DEX suppression test is commonly used to determine the negative feedback effects of DEX (a synthetic glucocorticoid) via the anterior pituitary GR to detect abnormalities with ACTH secretion through measurement of downstream cortisol production^22–24^. For greater sensitivity, this test is commonly combined with a subsequent CRH challenge, which allows for investigation of downstream stimulation of the adrenal glands by ACTH secretion from the anterior pituitary to release cortisol^22,24^. Briefly, pigs were weighed and housed in individual pens (2.4 × 1.8 m pen containing 1 nipple drinker and 1 feeder) 4 h prior to testing. On day 1 at 1900 h, a 2 mL blood sample (Sample 1) was collected from restrained pigs via jugular venipuncture (3 mL; serum; BD Diagnostics, Franklin Lakes, NJ, USA) and then all pigs were immediately injected intravenously with 20 μg/kg of DEX (Dexamethasone Injection, 2 mg/mL; # D-2953–04; Phoenix Pharmaceutical Inc., St. Joseph, MO, USA). On day 2 at 0700 h, a second 2 mL blood sample (Sample 2) was collected from each pig, and then the pigs were immediately injected intravenously with 1 μg/kg of bovine CRH (Bovine CRH C2671, 20 μg/mL mixed in 0.9% saline, Sigma Aldrich, Castle Hill, Australia). A third 2 mL blood sample (Sample 3) was collected from each pig at 0730 h and then all pigs were immediately euthanized via an intravenous injection of pentobarbital sodium at 1 mL/4.55 kg body weight per manufacturer recommendations (Fatal-Plus, Vortech Pharmaceuticals Ltd., Dearborn, MI, USA). Time to collect blood (from start of restraint to end of blood collection) was recorded (in seconds) and fitted as a linear covariate in the final cortisol analyses to account for the effects of restraint on circulating cortisol levels. Entire hypothalamus, pituitary, and adrenal samples were removed from all individual pigs, weighed, snap frozen in liquid nitrogen, and stored at −80 °C.

### Blood analyses

Serum was collected by centrifugation at 4 °C and 1900×*g* for 15 min, aliquoted and stored at −80 °C. Circulating serum cortisol concentrations were analyzed in duplicate using a commercially available radioimmunoassay kit (Cortisol RIA, MP Biomedicals LLC, Solon, OH, USA) according to manufacturer’s instructions. The intra-assay coefficient of variance was 5.4%, and the inter-assay coefficient of variance was 6.2%. Cortisol concentrations were used to calculate cortisol change from baseline (∆ CORT) for both the DEX suppression tests (Sample 2–Sample 1) and CRH challenge (Sample 3–Sample 2) in order to account for any potential differences in baseline cortisol levels or extra-adrenal glucocorticoid production not directly related to the HPA-axis response^25,26^.

### mRNA abundance

All collected samples were homogenized using a TissueRuptor (Qiagen, Germantown, MD, USA), 1 mL Trizol (Life Technologies Corporation, Carlsbad, CA, USA), and 200 μL chloroform (Sigma-Aldrich, St. Louis, MO, USA). Following a 2 min incubation, samples were centrifuged for 15 min at 12,000×*g* at 4 °C, and then the supernatant (total RNA) was pipetted and added to 1 equal volume of 70% ethanol. The total RNA solution was then transferred to spin columns and the total RNA was purified using the RNeasy Mini Kit (Qiagen, Germantown, MD, USA). The eluted total RNA was then analyzed for concentration and purity by spectrophotometry at 260/280 nm with a NanoDrop ND-1000 spectrophotometer (Thermo Fisher Scientific, Wilmington, DE, USA).

Single-strand complementary DNA was synthesized using 200 ng of total RNA sample in 20 μL reactions using a High Capacity complementary DNA Reverse Transcription Kit (Applied Biosystems, Foster City, CA, USA), according to manufacturer recommendations. Primers for real time polymerase chain reactions (PCR) were chosen based on previous research in swine and are listed in Table 1. Real time PCR reactions were performed in duplicate with Fast SYBR Green Master Mix (Applied Biosystems, Foster City, CA, USA) and tenfold diluted cDNA using a StepOnePlus Real-Time PCR System (Applied Biosystems, Foster City, CA, USA).

**Table 1:** Primers used for real time polymerase chain reactions.

Gene	Sequences 5’–3’ (forward/reverse)	Reference
*GR*	GATCATGACCGCACTCAACATG/TTGCCTTTGCCCATTTCAC	Poletto et al.^28^
*CRH*	CCGCCAGGAGGCACCCGAGAGG/GCCAAACGCACCGTTTCACTTC	Zhu et al.^29^
*POMC*	TCCGAGAAGAGCCAGACG/GGCTTTGGGGTCGGCTTC	Kalbe and Puppe^30^
*CRHR 1*	CTCATCTCCGCCTTCATCCT/CCAAACCAGCACTTCTCATT	Zhu et al.^29^
*CRHR 2*	CCGCAATGCCTACCGAGAAT/TCATCCAAAATGGGCTCGCA	Li et al.^31^
*MC2R*	CTGTGGTTTTGCCAGAGGAG/ GCAGGGAGAGGATGAACAGG	Murani et al.^32^

*GR* glucocorticoid receptor, *CRH* corticotropin-releasing hormone, *POMC* proopiomelanocortin, *CRHR 1* corticotropin-releasing hormone receptor 1, *CRHR 2* corticotropin-releasing hormone receptor 2, *MC2R* melanocortin 2 receptor.

Data were analyzed using the ∆∆CT method^27^ while considering *GAPDH* as the reference gene and the average of the IUTN pigs as the calibrator sample. Relative quantities, calculated as 2^−∆∆CT^, were used for the statistical analyses.

### Statistical analyses

All data were analyzed using the PROC GLIMMIX procedure in SAS 9.4 (Cary, NC, USA). Pig was considered the experimental unit for all analyses. In utero treatment (IUTN, IUHS), age (10 weeks, 15 weeks), and their interactions were included as fixed effects in all models. Sex was fitted in all models but was removed from the model if it was not significant. Litter of origin and repetition were included as random effects in all models. The assumptions of normality of error, homogeneity of variance, and linearity were performed in the SAS 9.4 software (Cary, NC, USA) based on visual inspection (e.g., scatter plot), Shapiro–Wilk test^33^ for verification of the normality assumptions, and the SAS homogeneity of variance test. Some data were either log- (hypothalamic CRH; anterior pituitary GR, CRHR 1, and CRHR 2) or square root- (∆ CORT and CRH; hypothalamic GR; anterior pituitary POMC; adrenal MC2R) transformed to meet normality assumptions when necessary. However, all transformed data have been back-transformed for ease of interpretation and all data are presented as least squares means (LSmean) ± standard error (SE). Restraint time (in seconds) was used as a linear covariate for the baseline cortisol and ∆ CORT analyses. A statistical significance between comparisons was defined when *P* ≤ 0.05 and a statistical trend was defined as 0.05 < *P* ≤ 0.10.

## Results

### Body and tissue weights

Body weight was greater (*P* < 0.01; 38.1 kg) at 15-weeks when compared to 10-weeks of age, regardless of in utero treatment (Table 2). Hypothalamus weight was greater (*P* = 0.04) for IUHS-15-week old pigs when compared to IUTN-15-week, IUHS-10-week, and IUTN-10-week old pigs, and increased for IUTN-15-week pigs when compared to IUTN-10-week old pigs (Table 2). An overall in utero treatment effect was observed where hypothalamus weight was greater (*P* = 0.03; 28.2%) for IUHS when compared to IUTN pigs (Table 2). Pituitary weight tended to be greater (*P* = 0.08; 61.5%) for 15-week versus 10-week-old pigs (Table 2). No other body weight or hypothalamus, pituitary, and adrenal gland weight differences (*P* > 0.05) or tendencies (*P* > 0.10) were detected with any comparison (Table 2).

**Table 2:** Effects of in utero heat stress (IUHS) and age on body weight and hypothalamus, pituitary, and adrenal gland weights in pigs.

Parameter	In utero treatment—age				SEM	*P*-value		
	IUTN-10-week	IUHS-10-week	IUTN-15-week	IUHS-15-week		IU	A	IU × A
*n*	12	12	12	12				
Body weight, kg	29.7	30.4	69.6	66.7	2.0	0.62	< 0.01	0.34
Hypothalamus, g	1.77^a^	1.90^ab^	2.63^b^	3.74^c^	0.38	0.03	0.11	0.04
Pituitary, g	0.13	0.13	0.22	0.20	0.02	0.62	0.08	0.55
Adrenal, g	0.49	0.35	0.53	0.52	0.14	0.24	0.63	0.29

Letters (^a, b, C^) indicate significant differences (*P* ≤ 0.05) within a row.

*IUTN* in utero thermoneutral, *IU* in utero treatment, *A* age.

### Cortisol analyses

#### Baseline cortisol

Baseline cortisol was reduced (*P* < 0.01) in 15-week-old (11.33 ± 3.43 ng/mL) versus 10-week-old (28.68 ± 3.34 ng/mL) pigs, regardless of in utero treatment or sex (data not presented). A sex effect was observed where male pigs had greater (*P* = 0.03) baseline cortisol (24.89 ± 3.31 ng/mL) when compared to female (15.12 ± 3.43 ng/mL) pigs (data not presented). No baseline cortisol differences were detected (*P* = 0.94) when comparing IUHS-10-week (26.95 ± 4.73 ng/mL), IUTN-10-week (30.41 ± 4.72 ng/mL), IUHS-15-week (9.89 ± 4.83 ng/mL), and IUTN-15-week (12.77 ± 3.43 ng/mL) old pigs (data not presented). No other differences (*P* > 0.05) or tendencies (*P* > 0.10) were detected for baseline cortisol with any comparison.

#### DEX suppression test

**Figure 1:**
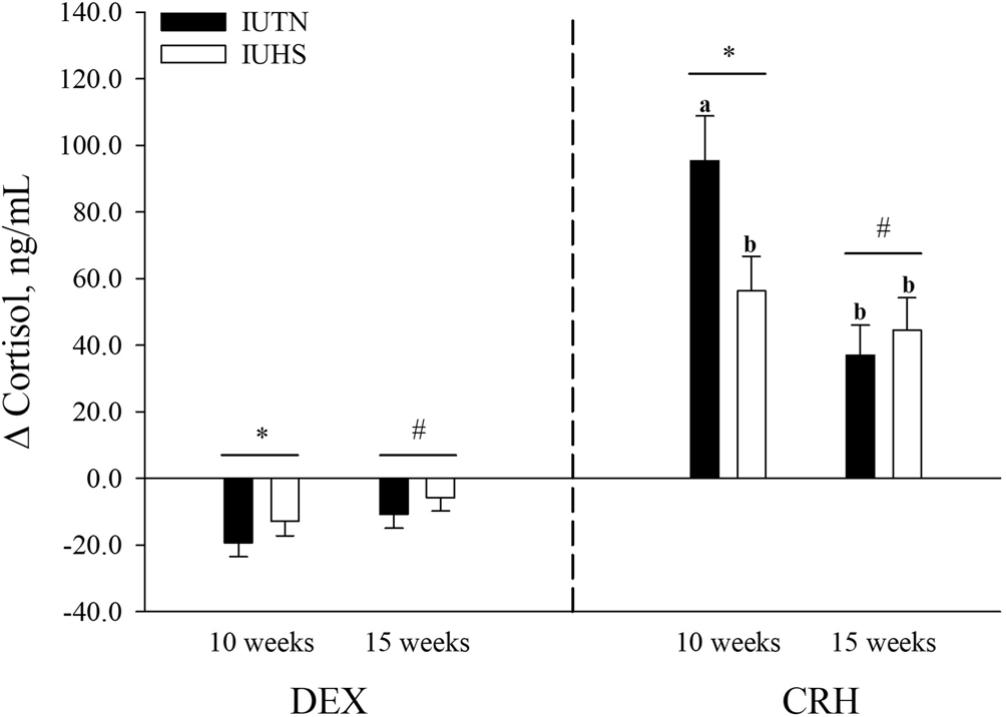
Effects of in utero heat stress (IUHS; *n* = 12 pigs per age category) or in utero thermoneutral (IUTN; *n* = 12 pigs per age category) conditions on the postnatal ∆ cortisol response of pigs following a dexamethasone (DEX) suppression test and a corticotrophin releasing hormone (CRH) challenge at 10 weeks and 15 weeks of age. Letters (a, b) indicate in utero treatment by age differences (*P* = 0.02). Symbols (*, #) indicate overall age statistical trends (0.05 < *P* ≤ 0.01). Data are presented as LSmeans ± SE.

The ∆ CORT tended to be reduced overall (*P* = 0.07) in 15-week-old (-8.26 ± 2.88 ng/mL) compared to 10-week-old (-15.98 ± 2.94 ng/mL) pigs (Fig. 1). No other differences (*P* > 0.05) or tendencies (*P* > 0.10) were detected for ∆ CORT in response to the DEX suppression test with any comparison (Fig. 1).

#### CRH challenge

An in utero treatment by age difference was detected where the ∆ CORT was reduced (*P* = 0.02) for IUHS pigs at 10 weeks of age (56.29 ± 10.45 ng/mL), IUHS pigs at 15 weeks of age (44.55 ± 9.68 ng/mL), and IUTN pigs at 15 weeks of age (37.02 ± 8.98 ng/mL), when compared to IUTN pigs at 10 weeks of age (95.39 ± 13.47 ng/mL; Fig. 1). Overall, ∆ CORT tended to be decreased (*P* = 0.07) in 15-week-old (40.70 ± 6.76 ng/mL) pigs when compared to 10-week-old (74.56 ± 8.69 ng/mL) pigs, regardless of in utero treatment (Fig. 1). No other differences (*P* > 0.05) or tendencies (*P* > 0.10) were detected for ∆ CORT in response to the CRH administration with any comparison (Fig. 1).

### mRNA abundance

#### Hypothalamus

**Figure 2:**
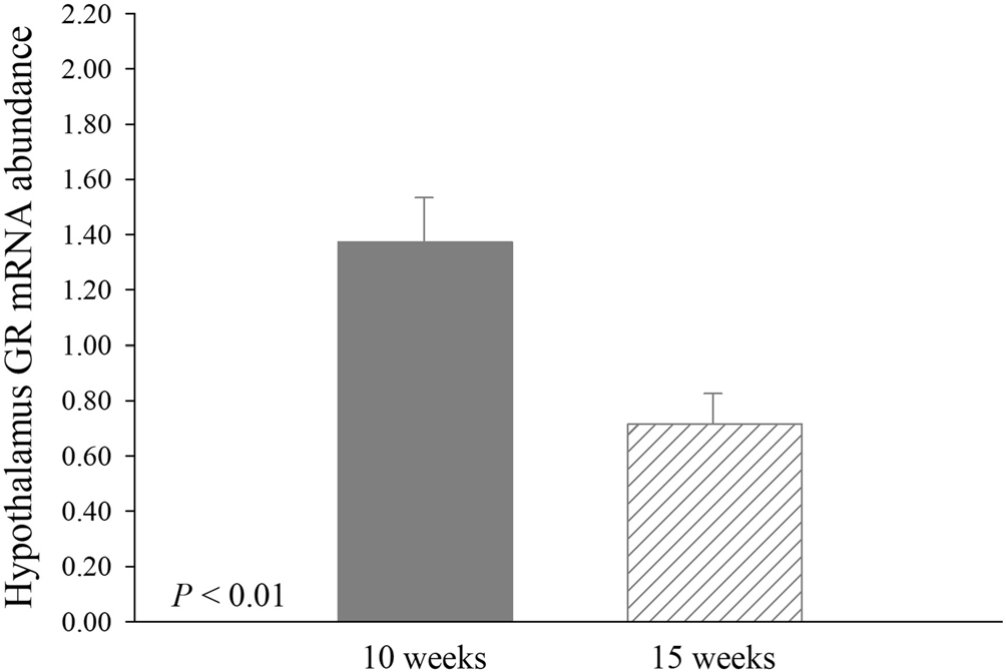
Effects of age [10 weeks (*n* = 24 pigs) and 15 weeks (*n* = 24 pigs)] on the mRNA abundance of glucocorticoid receptor (GR) in the hypothalamus of pigs. Data are presented as LSmeans ± SE.

Corticotropin releasing hormone mRNA abundance tended to be greater (*P* = 0.09; 47.2%) in IUHS pigs at 15 weeks of age when compared to IUTN pigs at 15 weeks of age, but no differences were detected when compared to IUHS and IUTN pigs at 10 weeks of age (Table 3). An overall decrease (*P* < 0.01) in GR mRNA abundance was detected in 15-week-old (0.72 ± 0.11) when compared to 10-week-old (1.37 ± 0.16) pigs, regardless of in utero treatment (Fig. 2). There were no further differences (*P* > 0.05) or tendencies (*P* > 0.10) detected for hypothalamic mRNA abundance with any comparison (Table 3, Fig. 2).

**Table 3:** Effects of in utero heat stress (IUHS) and age on the mRNA abundance of hypothalamic–pituitary–adrenal axis associated receptors and hormones in the hypothalamus, anterior pituitary, and adrenal glands of pigs.

Parameter	In utero treatment—age				SEM	*P*-value		
	IUTN-10-week	IUHS-10-week	IUTN-15-week	IUHS-15-week		IU	A	IU × A
*N*	12	12	12	12				
**Hypothalamus**								
GR	1.49	1.26	0.77	0.66	0.19	0.39	< 0.01	0.84
CRH	1.10^xy^	0.99^xy^	0.89^x^	1.31^y^	0.21	0.32	0.90	0.09
**Anterior pituitary**								
GR	1.83	1.62	0.40	0.39	0.25	0.81	0.03	0.76
POMC	1.26	1.20	1.02	1.15	0.23	0.87	0.47	0.64
CRHR 1	1.05	0.68	0.94	1.07	0.36	0.50	0.77	0.23
CRHR 2	1.09	1.56	0.86	0.93	0.49	0.42	0.58	0.55
**Adrenal**								
MC2R	1.30	1.07	1.29	0.82	0.20	0.11	0.38	0.41

Letters (^x, y^) indicate tendencies (0.05 < *P* ≤ 0.10) within a row.

*IUTN* in utero thermoneutral, *IU* in utero treatment, *A* age.

#### Anterior pituitary

**Figure 3:**
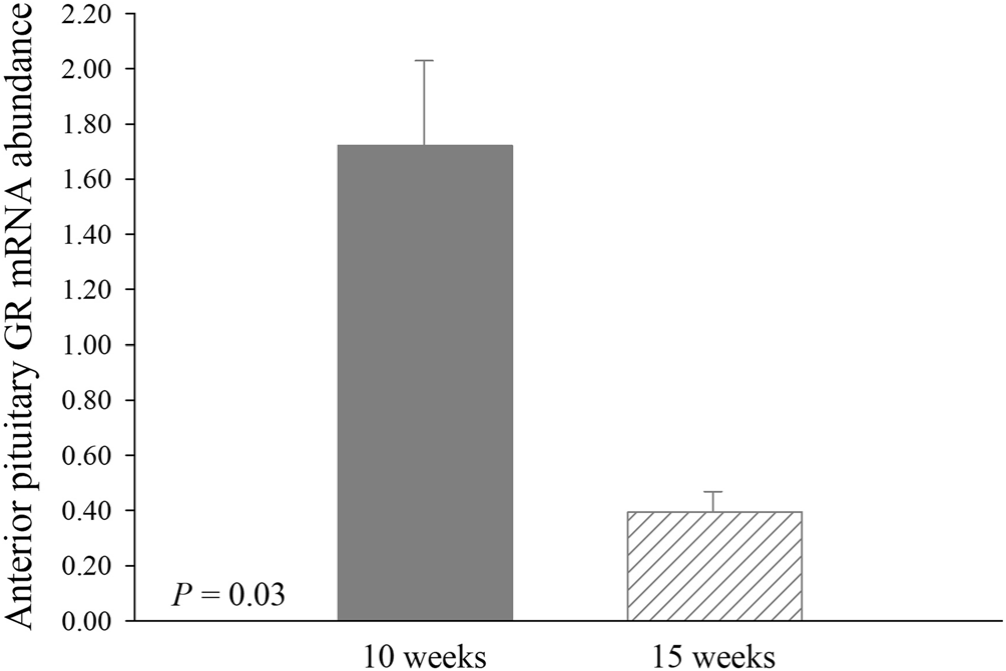
Effects of age [10 weeks (*n* = 24 pigs) and 15 weeks (*n* = 24 pigs)] on the mRNA abundance of glucocorticoid receptor (GR) in the anterior pituitary of pigs. Data are presented as LSmeans ± SE.

Glucocorticoid receptor mRNA abundance was decreased overall (*P* = 0.03) in 15-week-old (0.40 ± 0.07) when compared to 10-week-old (1.72 ± 0.31) pigs, regardless of in utero treatment (Fig. 3). There were no further differences (*P* > 0.05) or tendencies (*P* > 0.10) detected for anterior pituitary mRNA abundance with any comparison (Table 3, Fig. 3).

#### Adrenal

There were no differences (*P* > 0.05) or tendencies (*P* > 0.10) detected for adrenal mRNA abundance with any comparison (Table 3).

## Discussion

In utero heat stress negatively impacts multiple behavioral and physiological indicators of pig stress and welfare during postnatal life^1–8^. However, to our knowledge, there have been no efforts to elucidate mechanisms that control the postnatal stress response of IUHS pigs. A procedure previously used to evaluate HPA axis functionality in pigs is a DEX suppression test followed by a CRH challenge^22,23^. These tests are designed to detect abnormalities of ACTH secretion and/or altered negative feedback within the HPA axis^24^. In the present study, an in utero treatment by age difference was observed where IUHS-10-week, IUTN-15-week, and IUHS-15-week-old pigs had a decreased ∆ CORT response when compared to IUTN-10-week-old pigs in response to the CRH challenge. In this regard, we speculate that a reduced cortisol response within IUHS 10-week-old pigs could suggest a blunted ACTH response of the anterior pituitary to CRH stimulation when compared to IUTN-10-week-old pigs. Although limited information related to the postnatal HPA axis response to CRH challenges in IUHS-exposed mammals exist, previous research in transgenic mice models that overproduce CRH describe a desensitized HPA-axis response, whereby circulating ACTH and glucocorticoid levels do not immediately increase following a brief restraint stress^34^. However, when given only a CRH challenge (not a DEX/CRH challenge), no differences in the glucocorticoid response of transgenic mice that overproduce CRH have been observed relative to controls^35^. It is important to note however that the CRH test alone is a less sensitive method with less diagnostic utility to assess HPA-axis disturbances than the DEX/CRH test used in the present study^24^. In further support of the hypothesis that IUHS pigs may overproduce CRH under basal conditions, it was determined that hypothalamic mass was greater for IUHS versus IUTN pigs in the present study. This may have resulted in greater absolute CRH production due to an absolute increase in the number of CRH producing cells in the paraventricular nucleus relative to IUTN pigs. However, it should be mentioned that dissection of the hypothalamus can be a difficult process (i.e., some non-hypothalamic tissue may be inadvertently attached to the hypothalamus following dissection) and so this result should be re-confirmed in subsequent studies with pigs not subjected to a DEX/CRH test using histological analyses to quantify the number of CRH producing cells. Nevertheless, given the variety of impaired physiological and behavioral mechanisms implicated with an altered HPA axis response in transgenic mouse models^34^, these data may have negative implications for IUHS pigs.

Several postnatal physiological processes are impacted by IUHS that may have ties to HPA-axis functionality^1,2^. In utero HS in pigs results in more sensitive postnatal innate immune response^36^, hyperinsulinemia^7,8^, and greater adipose deposition^37^, all of which are known to be potential outcomes of HPA-axis dysfunction. Specifically, early developmental stress and HPA-axis dysfunction can lead to greater sensitivity of the innate immune system and increased pro-inflammatory cytokine production in humans^38,39^. In addition, recent research in pigs demonstrates that cortisol infusions cause a shift from an adaptive to an innate immune response^40^. Furthermore, glucocorticoids regulate metabolic homeostasis and excessive levels can cause hyperinsulinemia, insulin resistance, and increased fat deposition, which are all phenotypes previously observed in IUHS pigs^7,8,37^. Taken together, the altered HPA-axis response observed in IUHS pigs in the present study may be linked to the aforementioned phenotypes. However, further research should be conducted to confirm this hypothesis.

Corticotrophin-releasing hormone mRNA abundance tended to be increased in IUHS-15-week-old compared to IUTN-15-week-old pigs, and this may confirm that IUHS pigs have an over production of CRH in accordance with other animal models of an attenuated stress response^34^. However, this response did not coincide with an altered ∆ CORT in IUHS-15-week-old pigs when compared to IUTN-15-week-old pigs. It should be noted that pigs were artificially injected with CRH in close proximity to sample collection and the CRH challenge may have had an effect separate to the innate response due to IUHS. With this knowledge, it may be more appropriate in the future to examine the effect of mRNA abundance in relation to the HPA axis in the absence of a DEX suppression test and CRH challenge.

In response to the DEX suppression test, there was trend for reduced ∆ CORT in 15-week-old when compared to 10-week-old pigs suggesting a decrease in negative feedback inhibition. This response to aging has been observed in other species such as humans^41^ and rodents^42^ and is suggestive of HPA-axis dysregulation. Concurrently, the ∆ CORT in response to CRH challenge tended to be reduced in 15-week-old when compared to 10-week-old pigs in the present study. This response may be due to an increase in basal CRH production with advancing age because biomarkers of CRH expression are often increased in the hypothalamus of aging populations^43^. As a result, increased CRH production may result in a desensitized ∆ CORT response to CRH stimulation as previously hypothesized based upon transgenic mice research^34^. To our knowledge, this is the first time this has been described in pigs and these data suggest that aging related effects (most commonly investigated in mature animals or elderly humans) on the HPA-axis of pigs may be observed at a relatively young age.

Glucocorticoid receptor mRNA abundance was reduced in 15-week-old compared to 10-week-old pigs in both the hypothalamus and anterior pituitary, and this difference mirrors the decreased ∆ CORT response observed in both the DEX suppression test and CRH challenge. These similarities may provide insight into physiological mechanisms responsible for the decreased ∆ CORT in an older pig. Glucocorticoid receptors are known to be involved with maintenance of the circadian rhythm^44^. Changes in GR expression have been previously correlated with changes in cortisol response in relation to human depressive models but show the opposite effect of decreased GR expression^45^. To our best knowledge, this is the first time this difference has been observed in pigs and further research needs to be conducted to understand the mechanisms driving these gene expression changes.

The lack of differences in mRNA abundance in most comparisons must be considered in the context of the experimental design. Animals were euthanized relatively soon after receiving the CRH challenge as this was done to evaluate differences in mRNA abundance in relation to the DEX suppression testing and CRH challenge. However, this may have altered basal expression of these genes in the absence of any stressors, so further research would need to be conducted to specifically identify any differences in strictly basal expression of HPA axis associated receptors and hormones. One alternative could be to sample pigs that have not been exposed to a stress challenge to eliminate any effects that the DEX suppression test or CRH challenge may have had on mRNA abundance in this present study. In addition, considering that key genes interact in certain metabolic pathways to produce a stress response, performing a whole transcriptomics analyses (e.g., RNA-sequencing) as previously described in HS-exposed rodent models^46^ might contribute to identify additional genes that are differentially expressed across treatments and play a role in altered HPA axis function.

## Data Availability

All data can be made freely available from the corresponding author upon request.
